# Interim efficacy and preliminary survival outcomes of polatuzumab vedotin in combination for diffuse large B-cell lymphoma: a retrospective real-world study

**DOI:** 10.3389/fonc.2026.1847437

**Published:** 2026-09-04

**Authors:** Songshen Liu, Xia Zhao, Xue Shi, Hong Xu, Wei Wang, Lingjie Sun

**Affiliations:** Department of Hematology, The Affiliated Hospital of Qingdao University, Qingdao University, Qingdao, China

**Keywords:** antibody–drug conjugate, diffuse large B-cell lymphoma, interim efficacy, polatuzumab vedotin, real-world study

## Abstract

**Purpose:**

The standard first-line R-CHOP regimen achieves cure in only approximately 50% of patients. Polatuzumab vedotin (Pola) is a novel antibody-drug conjugate targeting the B-cell receptor component CD79b; however, evidence regarding its real-world efficacy remains relatively limited. This retrospective study aims to evaluate the clinical efficacy of Pola in combination therapy.

**Methods:**

A retrospective analysis was conducted on 140 patients with complete clinical records treated at the Affiliated Hospital of Qingdao University between September 2022 and September 2025. Among them, 114 patients received combination regimens containing Pola, while 26 patients received regimens without Pola. Given the limited follow-up duration, this study primarily assessed interim efficacy, with preliminary survival data also reported.

**Results:**

Among the 140 eligible patients, 4 deaths and 20 disease progressions occurred. The age range was 23–91, with a median age of 67; 45.0% were female. An ECOG performance status ≥2 was observed in 16.4% of patients, and 20.7% presented with B symptoms. Elevated LDH levels were found in 57.9% of patients. Ann Arbor stage III or IV disease was present in 65.0% of patients, and an IPI score >2 was recorded in 50.7%. Double-expressor lymphoma was diagnosed in 40.0% of cases. Bone marrow involvement was detected in 20.0% of patients, while 83.6% had lymph node extracapsular extension. Immunohistochemistry staining showed 36 patients (25.7%) with GCB subtype and 99 patients (70.7%) with non-GCB subtype. Median follow-up was 11 months (95% CI: 10.85–13.15). The objective response rate (ORR) was 94.4% in the Pola group and 70.0% in the non-Pola group (OR = 7.1, 95% CI: 2.0–27.2, P<0.001). Complete remission (CR) rates were 53.7% and 20.0%, respectively (OR = 4.6, 95% CI: 1.7–14.9, P = 0.002). The Pola group showed a statistically significant advantage in progression-free survival (PFS). Six-month PFS rates were 90.4% (95% CI: 84.8%–96.3%) for the Pola group and 76.9% (95% CI: 62.3%–94.9%) for the non-Pola group, while 12-month PFS rates were 87.2% (95% CI: 80.5%–94.6%) and 67.9% (95% CI: 51.7%–89.2%), respectively.

**Conclusion:**

Combination regimens containing Polatuzumab vedotin demonstrated a marked interim efficacy advantage, which may translate into potential long-term survival benefits.

## 1 Introduction

Diffuse large B-cell lymphoma (DLBCL) is the most common subtype of non-Hodgkin lymphoma, accounting for approximately 30% of cases (1). DLBCL exhibits pronounced clinical heterogeneity and is typically classified by cell-of-origin (COO) into germinal center B-cell-like (GCB) and non-germinal center B-cell-like (non-GCB) subtypes, with the GCB subtype generally associated with a more favorable prognosis while using R-CHOP regimen (2). Although the R-CHOP regimen (rituximab, cyclophosphamide, doxorubicin, vincristine, and prednisone) has been established as the standard first-line therapy and can achieve cure in a subset of patients, approximately 40% ultimately experience relapse or develop refractory disease (3). Common clinical manifestations include unexplained fever, night sweats, weight loss, and neurological involvement, which often progress rapidly and severely impair quality of life (4, 5). In relapsed/refractory cases, autologous hematopoietic stem cell transplantation may achieve disease control in selected patients, and CAR-T cell therapy has demonstrated therapeutic potential. Nevertheless, these approaches are constrained by limited applicability, significant toxicities, and reduced accessibility (6). Therefore, there is an urgent clinical need to develop more effective and safer therapeutic strategies.

CD79b, a critical component of the B-cell receptor complex, is broadly expressed on both normal and malignant B cells, including those in diffuse large B-cell lymphoma (DLBCL). It plays a pivotal role in B-cell survival and signaling, making it a highly attractive therapeutic target (7). Polatuzumab vedotin (Pola) is an antibody–drug conjugate (ADC) comprising an anti-CD79b monoclonal antibody covalently linked to the microtubule inhibitor monomethyl auristatin E (MMAE). Upon binding specifically to CD79b, the conjugate is internalized and releases the cytotoxic payload, thereby enabling selective targeting and elimination of B cells (8). Based on robust efficacy demonstrated in prospective studies, this agent was approved by the U.S. FDA in 2019 for the treatment of relapsed/refractory DLBCL (9). Nevertheless, real-world evidence regarding its combination use with other therapeutic agents remains limited and warrants further accumulation.

In this context, we analyzed clinical data from 140 patients with DLBCL treated with Pola-based combination regimens at the Affiliated Hospital of Qingdao University, including 114 patients who received Polatuzumab vedotin combination therapy, to assess the therapeutic efficacy of this strategy in routine clinical practice.

## 2 Methods

### 2.1 Patients

A retrospective study was conducted involving patients with DLBCL who received care at the Affiliated Hospital of Qingdao University between September 2022 and September 2025. Inclusion criteria: Patients with pathologist-confirmed untreated DLBCL who received at least one cycle of standard chemotherapy using the Pola-based regimen or R-CHOP regimen at this center between September 2022 and September 2025, had complete baseline clinical data available, and completed at least one PET/CT or CT imaging assessment of treatment response during the interim efficacy evaluation observation window and thereafter. Immunohistochemistry staining with the Hans algorithm was used to determine the COO (10). The Eastern Cooperative Oncology Group (ECOG) scoring system was used to evaluate patient performance status (11). The 2014 Lugano criteria were used for disease staging (12). Lesions with a maximum diameter of ≥10 cm were classified as bulky disease. And the IPI was calculated according to the standard definition (13).

### 2.2 Treatment

Of the 140 patients, 114 underwent Pola-containing combination therapy. Among these, 113 received Pola-R-CHP–based regimens, including 99 treated with standard Pola-R-CHP, 5 with Pola-R-miniCHP, and 9 patients who alternated between Pola-R-CHP and other regimens. The remaining 1 newly diagnosed patient switched to Pola-BR treatment after receiving 2 cycles of R-CDOP.

Due to local policies and patients’ financial constraints, 26 patients received therapy without Pola. 21 of them received standard R-CHOP, 3 received R-miniCHOP, 1 switched from R-CHOP to R2P after one cycle of R-CHOP, and 1 discontinued rituximab after one cycle of R-CHOP.

Pola-R-CHP regimen: Polatuzumab vedotin 1.8 mg/kg, rituximab 375 mg/m², cyclophosphamide 750 mg/m², and doxorubicin (or liposomal doxorubicin) 50 mg/m² intravenously on Day 1 of each cycle; prednisone 100 mg orally once daily on Days 1–5. This combination was administered for 6 cycles, followed by rituximab 375 mg/m² as monotherapy on Day 1 of cycles 7 and 8.

R-CHOP regimen: Rituximab 375 mg/m², cyclophosphamide 750 mg/m², doxorubicin (or liposomal doxorubicin) 50 mg/m², and vincristine 1.4 mg/m² (maximum 2 mg) intravenously on Day 1 of each cycle; prednisone 100 mg orally once daily on Days 1–5.

R2P regimen: Rituximab 375 mg/m² on Day 0 of each cycle; lenalidomide 25 mg on Days 1–21 of each cycle. Prednisone 100 mg on Days 1–5 of each cycle. One cycle consists of 28 days.

Pola-BR regimen: Polatuzumab vedotin 1.8 mg/kg, rituximab 375 mg/m², on Day 1 of each cycle. Bendamustine 90 mg/m² on Days 1-2 of each cycle.

### 2.3 Efficacy assessment and follow-up

Given the limited follow-up duration, interim efficacy evaluation was selected as the primary endpoint to assess short-term treatment outcomes of Pola-containing regimens. As an exploratory analysis, preliminary data on overall survival (OS) and progression-free survival (PFS) were also reported to provide early insight into long-term outcomes.

Interim efficacy assessments were performed when patients had completed half of their planned treatment courses. In clinical practice, assessments for some patients were conducted one to two cycles earlier or later than the midpoint of the scheduled therapy.

According to the 2014 Lugano criteria for staging and response assessment in Hodgkin and non-Hodgkin lymphoma, responses were evaluated using either PET/CT or CT (12). Treatment responses were classified as complete response (CR), partial response (PR), stable disease (SD), or progressive disease (PD). The overall response rate (ORR) was defined as the sum of CR and PR.

The follow-up cut-off date for this study was defined as the date of the patient’s last outpatient visit or hospitalization. Progression-free survival (PFS) was calculated as the interval from initiation of first-line chemotherapy to disease recurrence, progression, death from any cause, or the last follow-up visit. Overall survival (OS) was calculated as the interval from initiation of first-line chemotherapy to death or the last follow-up visit.

### 2.4 Statistical analysis

Statistical analyses were conducted using R software (version 4.3.3) and Microsoft Excel 2021. Baseline clinical characteristics were summarized using descriptive statistics. Intergroup differences were assessed using the chi-square test; Fisher’s exact test was applied when expected frequencies were less than five. Survival analyses were performed using the Kaplan–Meier method to generate OS and PFS curves, with log-rank tests applied to compare differences between groups. A P value < 0.05 was considered statistically significant.

## 3 Results

### 3.1 Baseline characteristics

A total of 140 eligible patients were enrolled, all confirmed to have CD20-positive DLBCL via tissue pathological immunohistochemistry, with one case representing transformation from follicular lymphoma. Four patients died, including two deaths occurring prior to efficacy assessment. Patient ages ranged from 23 to 91 years, with a median age of 67. There were 77 male patients (55.0%) and 63 female patients (45.0%). Twenty-three patients (16.4%) had an ECOG performance status score > 2. Twenty-nine patients (20.7%) presented with B symptoms. Elevated LDH levels were observed in 81 patients (57.9%). Ann Arbor stage III or IV disease was present in 91 patients (65.0%). An IPI score > 2 was observed in 71 patients (50.7%). Double-expressor lymphoma was identified in 56 patients (40.0%). Bone marrow involvement was noted in 28 patients (20.0%). Lymph node extracapsular extension occurred in 117 patients (83.6%). Twenty-two patients (15.7%) had bulky disease. Cytogenetic classification revealed 36 patients (25.7%) with GCB subtype and 99 patients (70.7%) with non-GCB subtype (**Table 1**).

**Table 1:** Clinical characteristics at baseline.

Characteristics	Total (n=140)		Pola-based regimen (n=114)		R-CHOP (n=26)	
Median age (range)	67	(23-91)	67	(31-91)	67.5	(23-85)
Gender (%)						
Male	77	55.0%	59	51.8%	18	69.2%
Female	63	45.0%	55	48.2%	8	30.8%
ECOG score (%)						
0-1	117	83.6%	95	83.3%	22	84.6%
≥2	23	16.4%	19	16.7%	4	15.4%
B symptom (%)						
No	107	76.4%	92	80.7%	15	57.7%
Yes	29	20.7%	20	17.5%	9	34.6%
Unknown	4	2.9%	2	1.8%	2	7.7%
LDH (%)						
Normal	59	42.1%	48	42.1%	11	42.3%
High	81	57.9%	66	57.9%	15	57.7%
AnnArbor stage (%)						
I-II	49	35.0%	38	33.3%	11	42.3%
III,IV	91	65.0%	76	66.7%	15	57.7%
IPI score (%)						
0~1	34	24.3%	28	24.6%	6	23.1%
2	35	25.0%	30	26.3%	5	19.2%
3	32	22.9%	25	21.9%	7	26.9%
4~5	39	27.9%	31	27.2%	8	30.8%
Cells of origin (%)						
GCB	36	25.7%	30	26.3%	6	23.1%
non-GCB	99	70.7%	80	70.2%	19	73.1%
Unknown	5	3.6%	4	3.5%	1	3.8%
DEL (%)						
Negative	76	54.3%	61	53.5%	15	57.7%
Positive	56	40.0%	47	41.2%	9	34.6%
Unknown	8	5.7%	6	5.3%	2	7.7%
Bone marrow involvement (%)						
No	112	80.0%	90	78.9%	22	84.6%
Yes	28	20.0%	24	21.1%	4	15.4%
Lymph node extracapsular extension (%)						
No	23	16.4%	15	13.2%	8	30.8%
Yes	117	83.6%	99	86.8%	18	69.2%
Bulky_disease (%)						
No	118	84.3%	95	83.3%	23	88.5%
Yes	22	15.7%	19	16.7%	3	11.5%

### 3.2 Interim efficacy

**Figure 1:**
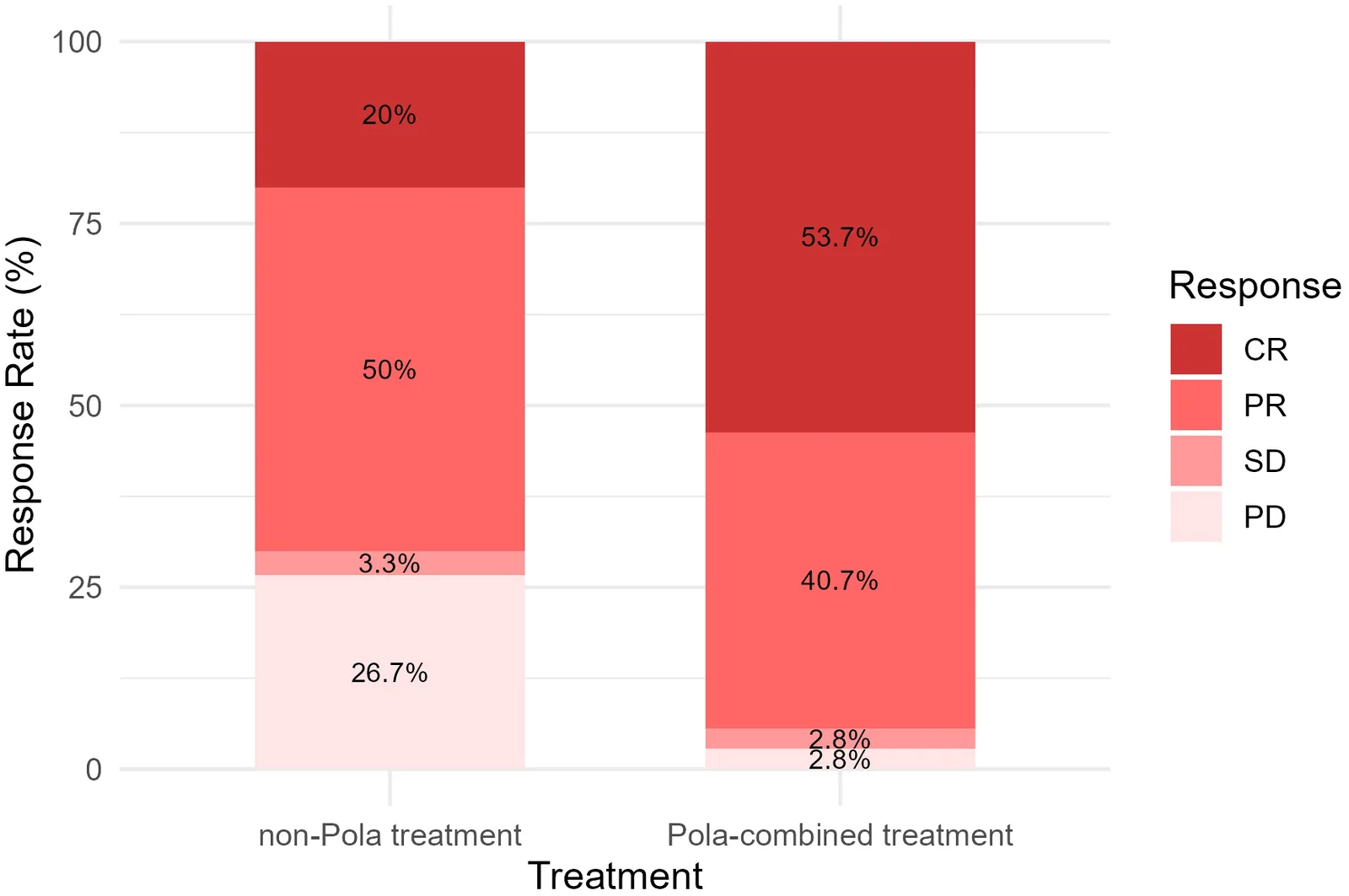
Efficacy of treatment in DLBCL patients.

Compared with the non-Pola group, the Pola-containing regimen demonstrated a higher remission rate (**Figure 1**). The objective response rates (ORR) were 94.4% and 70.0% (OR = 7.1, 95% CI: 2.0–27.2, P < 0.001) in the Pola and non-Pola groups, respectively, while complete response (CR) rates were 53.7% and 20% (OR = 4.6, 95% CI: 1.7–14.9, P = 0.002), respectively. To eliminate potential interference from cross-over, a sensitivity analysis excluding such patients was performed. The ORR in the Pola and non-Pola groups was 95.1% and 69.2% (OR = 8.4, 95% CI: 2.1–36.7, P < 0.001), respectively, with CR rates of 54.9% and 15.4% (OR = 6.6, 95% CI: 2.0–28.2, P < 0.001), respectively. These results were consistent with the primary analysis excluding cross-over.

**Figure 2:**
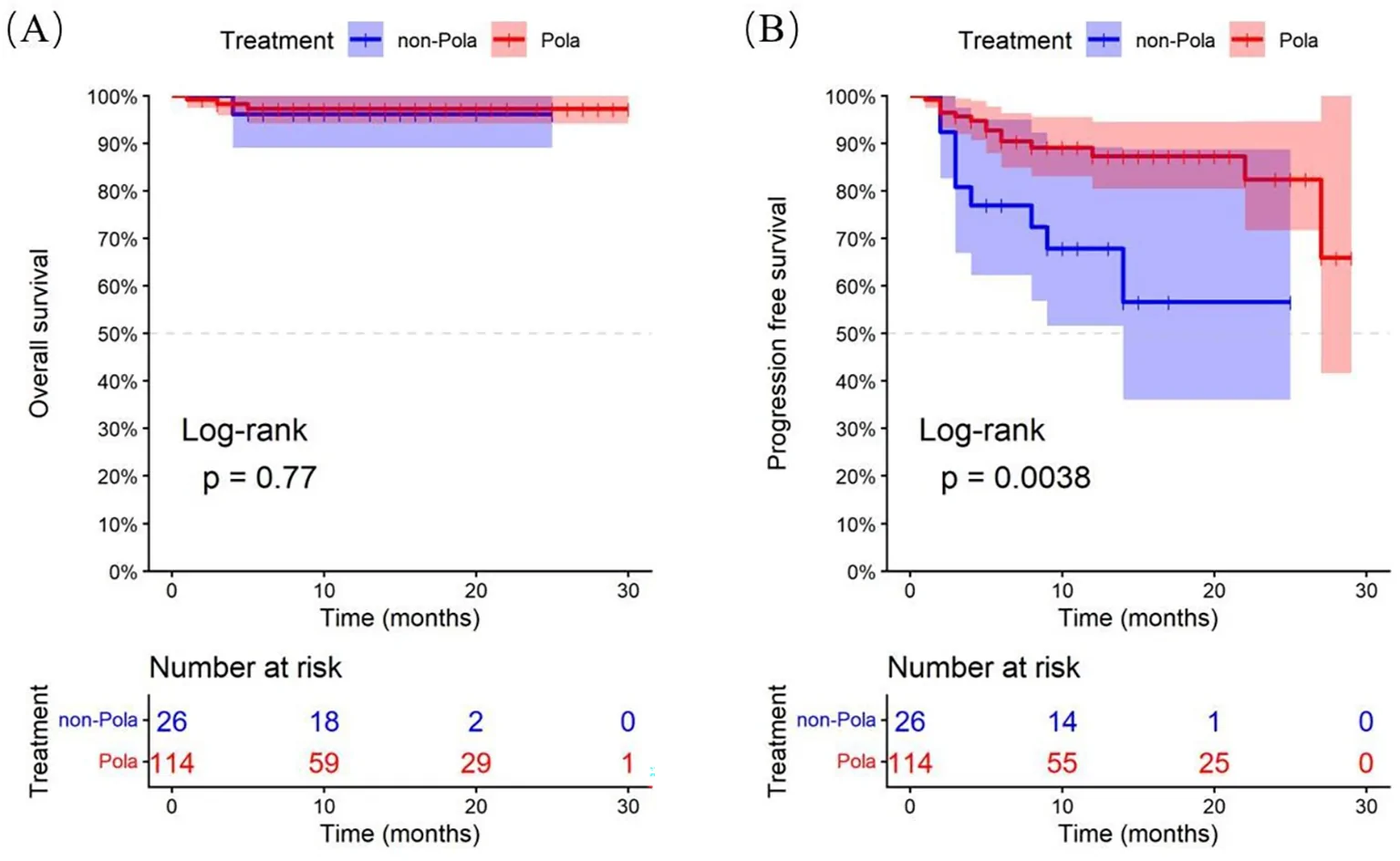
Kaplan-Meier curves. **(A)** OS. **(B)** PFS.

Subgroup analyses were conducted by dichotomizing various variables to compare efficacy within the Pola group. The ORR for GCB and non-GCB subtypes was 89.3% and 96.1% (P = 0.339), respectively, with CR rates of 50.0% and 57.9% (P = 0.620). The ORR for low-risk and intermediate/high-risk patients was 96.3% and 92.6% (P = 0.678), respectively, with CR rates of 64.8% and 42.6% (P = 0.034). The ORR for patients with normal versus elevated LDH levels was 97.8% and 91.9% (P = 0.237), respectively, with CR rates of 60.9% and 48.4% (P = 0.275). No statistically significant differences in response rates were observed among the subgroups (**Figure 2**).

### 3.3 Survival time

The median follow-up was 11 months (95% CI: 10.85–13.15). Among the 140 patients, 20 experienced relapse or progression, and 4 died, all due to complications.

Despite the limited follow-up period, an exploratory analysis of long-term outcomes was conducted. As most patients remained alive at the conclusion of follow-up, no statistically significant difference in overall survival (OS) was observed between the Pola and non-Pola groups (p=0.77). Progression-free survival (PFS) was significantly higher in the Pola group compared with the non-Pola group, with a log-rank test p-value of 0.0038. At the end of follow-up, neither median PFS nor median OS had been reached. The 6-month PFS rate was 90.4% (95% CI: 84.8%–96.3%) in the Pola group and 76.9% (95% CI: 62.3%–94.9%) in the non-Pola group. The 12-month PFS rates were 87.2% (95% CI: 80.5%–94.6%) in the Pola group and 67.9% (95% CI: 51.7%–89.2%) in the non-Pola group (**Figure 2**).

## 4 Discussion

DLBCL is a highly heterogeneous disease, and its prognosis is influenced by a variety of clinical and biological factors. Baseline characteristics such as age, LDH levels, and IPI scores collectively affect treatment outcomes, providing an important context for interpreting differences in treatment efficacy (14, 15). Against this background, based on a real-world retrospective cohort, this study demonstrated that within the predefined interim analysis window, the Pola group achieved significantly superior objective response rate (ORR) and complete response (CR) rate compared with the non-Pola group (94.4% vs. 70.0%, P<0.001; 53.7% vs. 20%, P = 0.002), indicating a clear short-term efficacy advantage for Pola combination therapy.

Polatuzumab vedotin is an anti-CD79b antibody-drug conjugate (ADC) that delivers the cytotoxic agent monomethyl auristatin E (MMAE) selectively to lymphoma cells. In 2019, on the basis of the favorable results of the GO29365 trial, the U.S. FDA granted accelerated approval to Pola in combination with bendamustine and rituximab for the treatment of relapsed/refractory DLBCL in the third-line setting and beyond (9). Subsequently, the POLARIX study further extended the application of Pola to the first-line setting; this trial demonstrated that the Pola-R-CHP regimen was superior to standard R-CHOP, thereby fundamentally transforming the therapeutic landscape of first-line DLBCL (16). In the ensuing years, multiple real-world studies have been published, corroborating the efficacy and safety of Pola across diverse patient populations. As real-world cohorts are typically more heterogeneous than those in controlled clinical trials, they offer supplementary evidence regarding the performance of Pola in routine clinical practice.

When we compare the results of this study with previously reported real-world data from Pola, we can identify some notable similarities and differences. Hotta et al. reported an ORR of 86.8% and a CR rate of 81.1% (17); Terao et al. reported an ORR of 90.8% and a CR rate of 75.7% (18); Zhao et al. reported an ORR of 93.0% and a CR rate of 86.8% (19); and the Asia subpopulation analysis of POLARIX study reported an ORR of 92.4% and a CR rate of 83.5% (20). Compared with the above results, the ORR in this study (94.4%) is comparable, but the CR rate (53.7%) is much lower. There may be multiple reasons for this discrepancy. First, there is a fundamental difference in the timing of efficacy assessment between our study and others. In the present study, efficacy was evaluated at a predefined mid-treatment window, when patients had completed only half of the planned therapeutic cycles. In contrast, the POLARIX trial and most real-world studies adopted end-of-treatment or best overall response as the primary efficacy endpoint. Response to anticancer therapy is a time-dependent and cumulative process; although the majority of responses may emerge during early cycles, a non-negligible proportion of additional responses can occur with continued treatment (21). For instance, in a study of patients with Waldenström macroglobulinemia receiving rituximab-containing regimens, approximately one-third of patients experienced further deepening of response after completion of therapy (22). Therefore, the discrepancy in assessment timing likely constitutes a major contributor to the substantially lower CR rate observed in our cohort compared with that in POLARIX and other published studies. In addition, significant differences in baseline patient characteristics were observed between the two cohorts. The POLARIX trial strictly enrolled treatment-naive patients aged 18–80 years, with a median age of 62 years, whereas the present study included nine patients over 80 years of age as well as relapsed/refractory cases, with a median age of 68 years. Studies have shown that DLBCL has a bad prognosis with advanced age (23). The proportion of patients with MYC/BCL2 double-expressor lymphoma in the Pola group of this study reached 41.2%, exceeding the 31.8% reported in POLARIX; this is noteworthy given that double-expressor status has consistently been associated with inferior response depth and long-term prognosis (24). The percentage of patients with Ann Arbor stage III–IV disease was 66.7%, higher than the 52.8% documented by Hotta et al. and the 64.9% reported by Terao et al. The rate of bone marrow involvement (21.1%) was also higher than the 10.4% reported by Zhao P., and prior evidence has identified bone marrow involvement as an independent adverse prognostic factor in DLBCL (25). Regarding treatment regimens, six patients in this study received a reduced-dose Pola-R-miniCHP regimen due to poor tolerability, and nine patients experienced treatment crossover as a result of regimen modifications; these factors may have collectively attenuated the observed efficacy. Finally, the relatively limited sample size of this study may also affect the precision of the efficacy estimates. Nonetheless, the real-world data indicate that the Pola-combination regimen achieves a high overall response rate even in a clinically more complex patient population, supporting its therapeutic potential across a broad spectrum of DLBCL cases.

In the clinical management of DLBCL, patients with the non-GCB cell-of-origin subtype consistently exhibit inferior outcomes compared with those with the GCB subtype under conventional R-CHOP therapy, a disparity extensively validated in both Eastern and Western populations (26, 27). Addressing this established prognostic factor, subgroup analyses within the Pola-treated cohort revealed no statistically significant differences in objective response rate (ORR) (p=0.339) or complete response (CR) rate (p=0.620) between the GCB and non-GCB subtypes. These findings, consistent with those reported by Cliff et al., suggest that Pola-based therapy may abrogate the prognostic gap between cell-of-origin subtypes observed under conventional treatment regimens (28).

We further evaluated long-term patient outcomes. Despite the wide enrollment period and limited follow-up, the Pola group demonstrated a significant improvement in progression-free survival (PFS). This is consistent with its interim efficacy advantage, suggesting that short-term responses may translate into potential long-term survival benefits. A LYSA study from the French DESCAR-T registry reported that, for patients eligible for subsequent CAR-T therapy or hematopoietic stem cell transplantation, the response to short-course bridging therapy directly influences their ultimate prognosis (29). Therefore, the short-term remission achieved with the Pola regimen not only secures a critical therapeutic window but may also confer long-term survival benefits by enhancing the effectiveness of bridging therapy.

Among all eligible patients, nine underwent cross-over due to protocol modifications prior to the interim analysis. To ensure the robustness of our findings, we adhered strictly to the intention-to-treat (ITT) principle in the primary analysis and performed sensitivity analyses excluding crossover cases, which produced results consistent with the primary analysis (30, 31). This confirms that our conclusions are not influenced by protocol adjustments commonly encountered in clinical practice.

This study is subject to several limitations. First, as a single-center retrospective analysis, it is inherently susceptible to selection and information biases, particularly with regard to treatment regimen selection and the consistency of the evaluation window. Second, the relatively limited sample size, especially after stratification into subgroups, constrained the statistical power and reliability of the subgroup analyses. Third, the follow-up duration was relatively short, with interim efficacy serving as the primary endpoint; survival data remain immature, as median progression-free survival and overall survival have not yet been reached, precluding an accurate assessment of the long-term survival benefits of Pola. Moreover, response assessment was based on either PET/CT or CT. Because not all patients underwent uniform PET/CT-based evaluation, some degree of variability in response classification cannot be excluded, and this should be considered an additional methodological limitation of this study. In addition, this study has a broad age span and thus some age heterogeneity, therefore a residual confounding effect of age cannot be entirely excluded. Finally, although the GCB subtype encompasses a small subset of double-hit/triple-hit high-grade B-cell lymphomas with an exceedingly dismal prognosis, virtually all patients in this study lacked fluorescence *in situ* hybridization (FISH) testing, owing to limited institutional availability and patients’ financial constraints (32). Consequently, we were unable to further stratify GCB patients harboring these high-risk genetic abnormalities. Future investigations should aim to expand the sample size through multicenter collaboration to enhance the representativeness and generalizability of the findings. Additionally, Cox proportional hazards models should be constructed to evaluate the independent effect of Pola on PFS and OS after adjusting for potential confounders. Furthermore, Broader implementation of FISH testing is warranted to identify double-hit and triple-hit lymphomas, which are associated with a particularly poor prognosis.

## Data Availability

The original contributions presented in the study are included in the article/supplementary material. Further inquiries can be directed to the corresponding authors.
